# The DROP‐AD project: AD biomarkers from dry blood spots

**DOI:** 10.1002/alz70856_102258

**Published:** 2025-12-25

**Authors:** Hanna Huber, Wagner Scheeren Brum, Laia Montoliu‐Gaya, Anna Dittrich, Silke Kern, Millie Sander, Anja Hviid Simonsen, Anne Corbett, Juan Fortea, Sebastian Palmqvist, Barbara Borroni, Oskar Hansson, Mercè Boada, Xavier Morató Arús, Kaj Blennow, Henrik Zetterberg, Nicholas J. Ashton

**Affiliations:** ^1^ University of Gothenburg, Gothenburg, Sweden; ^2^ Department of Psychiatry and Neurochemistry, Institute of Neuroscience and Physiology, The Sahlgrenska Academy, University of Gothenburg, Mölndal, Sweden; ^3^ Institute of Physiology and Neuroscience, Sahlgrenska Academy, University of Gothenburg, Gothenburg, Sweden; ^4^ Region Västra Götaland, Sahlgrenska University Hospital, Department of Neuropsychiatry, Gothenburg, Sweden; ^5^ University of Exeter, Exeter, United Kingdom; ^6^ Danish Dementia Research Centre, Dept. of Neurology, Copenhagen University Hospital ‐ Rigshospitalet, Copenhagen, Denmark, Denmark; ^7^ Sant Pau Memory Unit, Hospital de la Santa Creu i Sant Pau, Biomedical Research Institute Sant Pau, Barcelona, Spain; ^8^ Clinical Memory Research Unit, Department of Clinical Sciences Malmö, Lund University, Lund, Sweden; ^9^ University of Brescia, Brescia, Lombardy, Italy; ^10^ Memory Clinic, Skåne University Hospital, Malmö, Sweden; ^11^ Ace Alzheimer Center Barcelona – International University of Catalunya (UIC), Barcelona, Spain; ^12^ Ace Alzheimer Center Barcelona‐Universitat Internacional de Catalunya, Barcelona, Spain; ^13^ University of Gothenburg, Mölndal, Sweden; ^14^ Clinical Neurochemistry Laboratory, Sahlgrenska University Hospital, Mölndal, Västra Götalands län, Sweden; ^15^ Banner Alzheimer's Institute, Phoenix, AZ, USA

## Abstract

**Background:**

In less than a decade, blood‐based biomarkers for Alzheimer's disease (AD) have evolved from a promising research tool to a valuable clinical asset. Increased accessibility to biomarkers such as pTau217 could significantly enhance population screening, epidemiological studies, and the inclusion of underrepresented groups in dementia research. The DROP‐AD project aims to streamline blood sample collection by introducing an alternative method that overcomes challenges associated with traditional venous draws while preserving biomarker integrity and value.

**Method:**

We recruited 344 participants (70.8 [11.7] years; 167 females [53.4%]) from eight centers, obtaining paired venous EDTA plasma and dried plasma spots (DPS) from capillary finger‐stick collection. Capillary samples were shipped to Gothenburg, Sweden, without temperature control, extracted using a custom protocol, and analyzed with the ALZpath pTau217 Simoa assay. Cerebrospinal fluid (CSF) biomarkers were available for 151 individuals. Participants were classified as Aβ‐positive (Aβ+) based on CSF Aβ42/pTau181 ratio. Cognitive assessments (MMSE and/or CDR‐Global) were conducted for all participants. Statistical analyses included Spearman correlations, linear models, receiver operating characteristic (ROC) curve analysis, and evaluation of diagnostic performance using predefined cut‐offs.

**Result:**

Capillary DPS pTau217 showed a strong correlation with venous plasma pTau217 across all cohorts (rs=0.74, 95% CI: 0.68–0.79; *p* <0.001). It was significantly associated with MMSE scores (r=‐0.37; *p* <0.0001) and age (r=0.33, 95% CI; *p* <0.0001), mirroring the venous plasma results. Capillary DPS pTau217 was markedly elevated (+198%) in individuals with AD pathology, with a discriminative accuracy of 0.863 (95% CI: 0.81–0.92). For NfL (r=0.83, 95% CI: 0.74–0.89) and GFAP (r=0.773, 95% CI: 0.71–0.82), we observed strong correlations between capillary DPS and venous plasma measurements. In a subgroup of 31 participants with Down syndrome, capillary and venous blood biomarker levels were significantly correlated (GFAP: r=0.629, 95% CI: 0.35–0.81; pTau217: r=0.875, 95% CI: 0.50–0.97). Further ancillary studies will assess the reproducibility of DROP‐AD across independent cohorts and evaluate its performance in self‐guided collection settings.

**Conclusion:**

This study demonstrates the feasibility of detecting and accurately quantifying AD biomarkers from capillary blood. The results suggest that this simple and temperature‐independent approach could facilitate the identification of individuals at high risk for AD pathology, broadening access to biomarker testing in research settings.

